# Supplementary material for: The interactome of intact mitochondria by cross-linking mass spectrometry provides evidence for coexisting respiratory supercomplexes
Source: Mol Cell Proteomics. 2017 Dec 8;17(2):216–32. doi: 10.1074/mcp.RA117.000470 (PMC5795388; doi:10.1074/mcp.RA117.000470)
Supplement: Supplemental Data [file supp_RA117.000470_133922_0_supp_23978_fzffwf.zip › spectra_annotation/mito_DR_spectra_annotation/318-1-14-1-1-1.pdf]

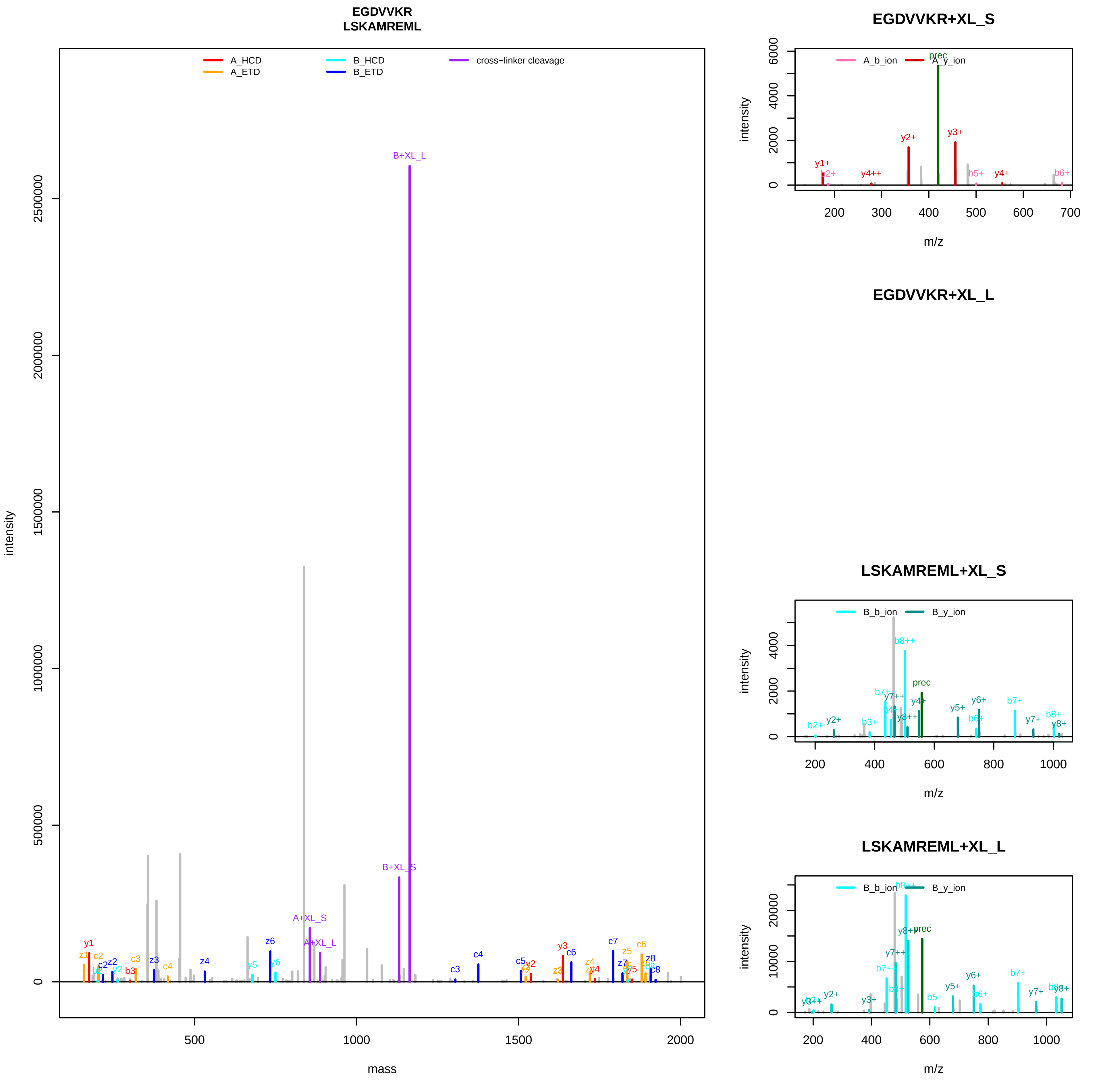

EGDVVKR
LSKAMREML
A_HCD
A_ETD
B_HCD
B_ETD
cross−linker cleavage
B+XL_L
B+XL_S
A+XL_S
A+XL_L
y1 z1 c2 b2 c2 z2 y2 b3 c3 z3 c4 z4 y5 z6 y6 c3 c4 c5 z2 y2 z3 y3 c6 z4 z4 y4 c7 z7 z5 y5 c6 z8 c8
intensity
mass
0
500000
1000000
1500000
2000000
2500000
500
1000
1500
2000
EGDVVKR+XL_S
A_b_ion
A_y_ion
prec
y1+ b2+ y4++ y2+ y3+ b5+ y4+ b6+
intensity
m/z
0
2000
4000
6000
200
300
400
500
600
700
EGDVVKR+XL_L
LSKAMREML+XL_S
B_b_ion
B_y_ion
b8++ prec b7++ y7++ y4+ b4+ y8++ b2+ y2+ b3+ y5+ y6+ b6+ b7+ y7+ b8+ y8+
intensity
m/z
0
2000
4000
200
400
600
800
1000
LSKAMREML+XL_L
B_b_ion
B_y_ion
b8++ y8++ prec y7++ b7++ b3++ y3++ y2+ y3+ b4+ b5+ y5+ y6+ b6+ b7+ y7+ b8+ y8+
intensity
m/z
0
10000
20000
200
400
600
800
1000
